# Sharp Phylogeographic Breaks and Patterns of Genealogical Concordance in the Brine Shrimp *Artemia franciscana*

**DOI:** 10.3390/ijms10125455

**Published:** 2009-12-18

**Authors:** Stefania Maniatsi, Ilias Kappas, Athanasios D. Baxevanis, Theodora Farmaki, Theodore J. Abatzopoulos

**Affiliations:** 1 Department of Genetics, Development & Molecular Biology, School of Biology, Aristotle University of Thessaloniki, 541 24, Greece; E-Mails: smaniats@bio.auth.gr (S.M.); ikappas@bio.auth.gr (I.K.); tbaxevan@bio.auth.gr (A.D.B.); 2 Institute of Agrobiotechnology, Centre for Research and Technology, Thermi, 570 01, Thessaloniki, Greece; E-Mail: mfarmaki@certh.gr

**Keywords:** mitochondrial DNA, zooplankton, endemism, dispersal, superspecies, coalescence, Andes, incipient speciation

## Abstract

Genealogical concordance is a critical overlay of all phylogenetic analyses, irrespective of taxonomic level. To assess such patterns of congruence we have compiled and derived sequence data for two mitochondrial (16S rRNA, COI) and two nuclear (ITS1, p26) markers in 14 American populations of the hypersaline branchiopod *Artemia franciscana*. Cladistic analysis revealed three reciprocally monophyletic mitochondrial clades. For nuclear DNA, incomplete lineage sorting was evident presumably as a result of slower coalescence or male-mediated dispersal. Our findings capture the genealogical interval between gene splitting and population divergence. In this sense, strong indications are provided in favour of a superspecies status and ongoing speciation in *A. franciscana*.

## 1. Introduction

Of the many significant contributions stemming from the field of phylogeography, the recognition of distinct phylogroups within and between closely related species holds a prominent position. Tightly linked to population demography and historical biogeography, the geographic patterning of intraspecific lineages has been the linchpin between population genetics and phylogeny.

The presence of intraspecific mitochondrial phylogenetic discontinuities provides multiple signals on population genealogy, depending on the geographic representation of mitochondrial lineages and their levels of divergence (see [1]). Normally, large phylogenetic splits provide evidence for long-term extrinsic barriers to genetic exchange. The observed genetic differences reflect the accumulation of *de novo* mutations in lineages that diverged in allopatry. Less prominent gaps imply contemporary restraints on gene flow, at least *via* females. One important aspect of the previous outcomes and their variations is whether the observed phylogenetic splits are also registered across several loci, ideally in a combination of mitochondrial and unlinked nuclear genetic markers. Genealogical concordance is a critical conceptual overlay of all phylogenetic inferences. In a phylogeographic context, while concordance within a gene provides statistical significance for gene-tree clades, a similar congruence of multiple genes within a species establishes fundamental phylogenetic partitions at the population or species level [2]. Striking examples of such agreement include the populations of the North American killifish *Fundulus heteroclitus* [3] and those of the Californian coastal copepod *Tigriopus californicus* [4,5].

Although a great variety of taxa have been surveyed for genealogical patterns on a regional scale (see [2]), organisms inhabiting the continental aquatic realm have presumably benefited the most of such approaches, as a result of their idiosyncratic biology and physical structure of their environments. Continental zooplanktonic organisms exhibit remarkably wide geographical distributions [6,7]. They typically inhabit small to medium size ponds and pools or larger lakes, covering the whole spectrum of water chemistry (freshwater to brackish to hypersaline). Despite the island-like nature and isolation of their habitats [8], continental zooplankters show marked cosmopolitanism, presumably due to passive transport of their diapausing propagules through wind or waterfowl [9]. This, coupled with their striking morphological uniformity, led to early suggestions [10] that continental zooplankters would be characterized by substantial levels of gene flow and, thus, their gene pools would be nearly panmictic. However, over the last twenty years or so, the introduction of molecular marker assays has overturned this notion by providing evidence for marked endemicity and significant intraspecific genetic diversity (see [7] and references therein). Consequently, views on aquatic invertebrates in general have shifted towards provincialism as the default biogeographic hypothesis, frequently accounted for by allopatric divergence [6,7,11,12]. In genealogical terms, empirical data span the entire continuum, from sharply demarcated phylogroups [4,13] to cryptic species assemblages [14–18]. A general, yet not all-inclusive (*i.e.*, absence of physical barriers), explanation of phylogeographic patterns in continental zooplankton has been provided by De Meester *et al*. [19] implicating priority effects and strong local adaptation in the severe reduction of effective gene flow among resident populations. Thus, inferred genealogies reflect historical colonization patterns rather than low contemporary gene flow.

In the present study, we explore the genetic architecture of the anostracan branchiopod *Artemia franciscana. Artemia* is a typical zooplankter of saline lakes, ponds, and solar saltworks worldwide [20,21]. These biological communities are considerably diverse in terms of physico-chemical parameters and seasonality [21,22] and impose severe physiological demands which the brine shrimp overcomes through an array of adaptations in parturition (oviparity *versus* ovoviviparity), mode of reproduction (sexuality *versus* parthenogenesis), and life cycle (cryptobiosis in the form of diapausing encysted embryos) (see [23]). Currently, six bisexual species and a heterogeneous assemblage of obligate parthenogens are recognized within the genus [11,24]. The sexual *A. franciscana* is probably the most extensively studied species. It is endemic to the New World, but permanent and temporal populations also exist worldwide due mainly to anthropogenic introductions of different strains [23,25]. Recent evidence indicates that the lineage leading to *A. franciscana* diverged from its sister group (comprised of parthenogens and Asian bisexual species, namely *A. sinica*, *A. tibetiana*, and *A. urmiana*) approximately 30 million years ago (mya) [11]. It then radiated over the whole American continent in a varied suite of environments, forming a diverse and differentiated group of populations. This differentiation is evident in an array of attributes including genetic polymorphisms, temperature/salinity tolerances, reproductive and life-span characteristics, and strain-specificity in diapause deactivation (see [26] and references therein). Owing to its overall distinctive biology, *A. franciscana* is regarded as a “superspecies” in the process of incipient speciation [27]. Despite being a model-system in *Artemia* research and having peculiar genome integrity [12], *A. franciscana* lacks dedicated phylogeographic analyses using molecular data. In this work, we address this issue by examining patterns of population differentiation and genealogical concordance using sequence data from two mitochondrial (16S, COI) and two nuclear (ITS1, p26) molecular markers.

## 2. Results

### 2.1. Mitochondrial Loci

We have sampled 14 *A. franciscana* populations distributed in North and South America and obtained sequence information from a total of four mitochondrial and nuclear loci. The 16S rRNA sequences ranged in length from 455 to 458 bp, with a total of 19 parsimony-informative sites. Base frequencies were homogeneous across taxa (*χ*^2^ = 13.22, *df* = 60, *p* = 0.99). Similarly, inspection of plots between transitions/transversions and corrected distances (TrN + G, *α* = 0.20) revealed no signs of substitution saturation (data not shown). The *g*_1_ value (*g*_1_ = −0.659, *p* < 0.05) indicated that the tree-length distribution was significantly more left-skewed than expected from random data, thus suggesting the presence of phylogenetic signal. Pairwise corrected distances for the ingroup ranged from zero to 3.7%. Distances to outgroup (*A. salina*) ranged from 45.3 to 62.8%.

Similar patterns were also obtained for the COI data set (582 bp, 31 parsimony-informative sites). Pairwise ingroup distances (HKY + G, *α* = 0.27) ranged from zero to 4.7%, while ingroup-to-outgroup divergence values ranged from 39.4 to 44.1%.

### 2.2. Nuclear Loci

A similar picture regarding data composition (base frequencies) and quality (substitution saturation, presence of phylogenetic signal) was obtained for both nuclear loci. For ITS1 (ingroup sequence length 1165–1182 bp), base frequency homogeneity could not be rejected (*χ*^2^ = 6.82, *df* = 60, *p* = 0.99), while the tree-length distribution was significantly left-skewed (*g*_1_ = −0.520, *p* < 0.05). Intraspecific and interspecific (to *A. salina*) distance estimates (HKY + G, *α* = 0.37) ranged from zero to 4.7% and from 23.5 to 28.1%, respectively.

In a previous study using the second intron of p26 as a discriminant marker in *A. franciscana*, Maniatsi *et al*. [29] identified a distinct pattern of allelic polymorphism between populations from San Francisco Bay (SFB) and Great Salt Lake (GSL). To ensure adequate resolution of the allelic diversity within our data set we preliminary screened several individuals (25–40) from each population. Three different p26 alleles were recovered as gauged by visualization of PCR-amplified fragments under UV light: the most common p26 allele corresponds to a fragment of ~1500 bp, the second allele corresponds to a fragment of ~2000 bp while the third bears a length of ~1400 bp. Populations GSL, CEJ, and CON were polymorphic for these three alleles and sequencing was performed in selected individuals (Table 1). Ingroup diversity estimates for p26 (HKY + G, *α* = 0.58) varied from a minimum of 0.1% to a maximum of 15.2%. Highest values were observed for the haplotypes corresponding to ~1500 bp (0.4–14.8%) while for the ~2000 and ~1400 bp alleles genetic diversities were 0.1–7.5% and 0.2–3.4%, respectively.

### 2.3. Phylogenetic Inference and Population Structure

**Figure 1.**
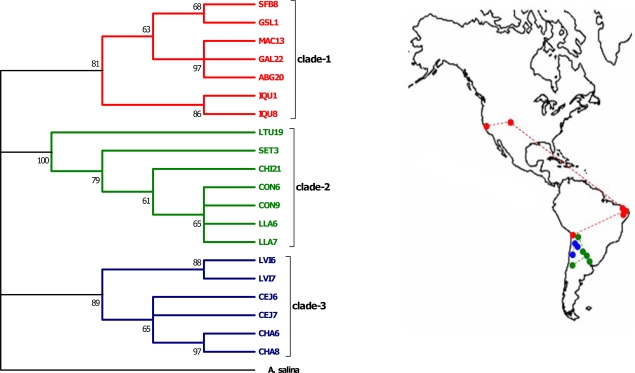
Consensus maximum parsimony phylogeny (50% majority rule of 124 most parsimonious trees, tree length = 230) of concatenated 16S and COI *A. franciscana* haplotypes. Values at nodes indicate bootstrap support based on 1000 pseudoreplicates. The geographic distribution of the recovered clades (connected by dashed lines for illustration purposes only) is shown on the map. The phylogeny is rooted with *A. salina*.

The pairwise 16S and COI genetic distance matrices were significantly correlated (Mantel test: *Z* = 0.88, *r* = 0.99, *p* < 0.001, 1000 randomizations). Three well-differentiated clades with high nodal support were recovered with the mitochondrial DNA data. The same topology was also recovered when considering third codon positions for the COI gene (data not shown). The maximum parsimony analysis of the concatenated genes (16S + COI) recovered 124 most parsimonious trees (tree length = 230). The consensus phylogeny is shown in Figure 1. The first clade consists of the North American populations SFB, GSL, the Brazilian feral populations MAC, GAL, ABG, and the northernmost Chilean population of IQU. In the second clade, the Chilean populations of CON and LLA are clustered with all three Argentinean representatives (LTU, SET, CHI). The third clade consists of the remaining Chilean populations (LVI, CEJ, CHA). Analysis of population structure on the basis of the mitochondrial DNA topology revealed a strong pattern of population differentiation. The *K*_ST_ statistic was 0.74 (*p* < 0.001, 10,000 randomizations) meaning that sequence differences segregate according to clade origin. In addition, there was no evidence for an isolation-by-distance pattern as judged by the lack of correlation between genetic and geographic distances, excluding the three Brazilian feral populations (Mantel test: *Z* = 130.43, *r* = 0.21, *p* = 0.13).

**Figure 2.**
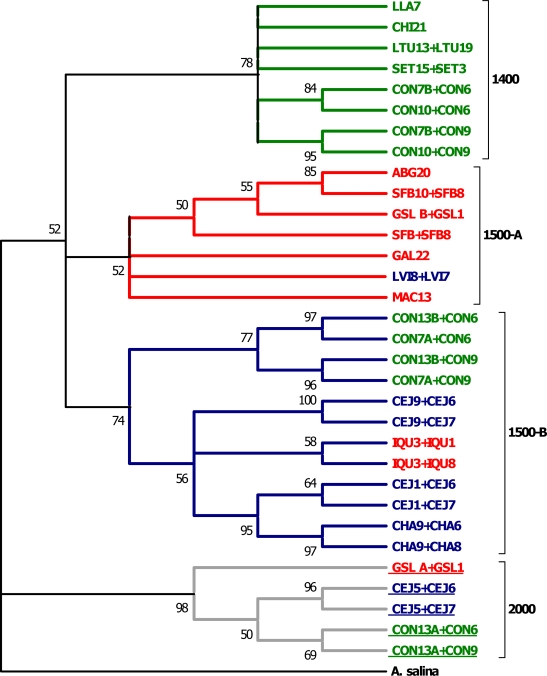
Consensus maximum parsimony phylogeny (50% majority rule of 38 most parsimonious trees, tree length = 1224) of concatenated p26 and ITS1 A. franciscana haplotypes. Values at nodes indicate bootstrap support based on 1000 pseudoreplicates. The order of concatenation is denoted as p26 + ITS1. Designations lacking a plus sign indicate concatenation of sequences from the same individual. The recovered groups (1400, 1500-A, 1500-B, 2000) are used as tags for the observed p26 alleles (see text). Colors (green, red, blue) correspond to the respective mitochondrial clades (see Figure 1). Haplotypes of the distinct 2000 group (gray) are underlined. The phylogeny is rooted with A. salina.

For the combined nuclear data, significant correlation was also evident between the ITS1 and p26 genetic divergence matrices (Mantel test: *Z* = 2.85, *r* = 0.98, *p* < 0.05, 1000 randomizations). The inferred phylogeny using maximum parsimony (38 equally parsimonious trees, tree length = 1224) is shown in Figure 2. Four groups are recovered corresponding to the different lengths of the p26 alleles (1400, 1500, and 2000 bp) with the 1500 bp group splitting further in two clusters. The most divergent group is the one bearing the 2000 bp haplotypes: it shares a more distant common ancestor with the others groups (see Figure 2), with distance values of 8.2% (2000 *versus* 1500) and 7.2% (2000 *versus* 1400), while 1500 *versus* 1400 equals 4.8%. Interestingly, the 2000 bp group retains a similar phylogenetic structure with that of the entire mitochondrial DNA tree (contains representatives from the three mitochondrial DNA clades). The remaining three groups (1400, 1500-A, 1500-B) are characterized by various degrees of cohesion and concordance to the recovered mitochondrial clades. Of particular importance are the LVI and IQU denoted haplotypes whose closest relatives reside in different mitochondrial and nuclear clades. It should be emphasized that allele lengths (1400, 1500, 2000) are used only as tags in the nuclear phylogeny; the resulting groups are based solely on sequence differences/similarities since gaps were considered as missing data.

## 3. Discussion

We have employed a battery of mitochondrial (16S, COI) and nuclear (ITS1, p26) markers to investigate patterns of genealogical concordance in the hypersaline branchiopod *A. franciscana*. The current study is the first dedicated attempt to explore the historical phylogeography of this model anostracan using sequence data from several loci. Although limited by current standards, our data set provides strong evidence for an advanced state of phylogenetic congruence across loci in the investigated lineages.

### 3.1. Mitochondrial Genealogy

The inferred mitochondrial genealogy is a typical example of pronounced phylogenetic gaps between allopatric lineages. The presence of three reciprocally monophyletic mitochondrial phylogroups (clades 1–3, see Figure 1) with statistically significant support is reminiscent of phylogeographic discontinuities modulated by long-term extrinsic barriers to genetic exchange or low gender-specific dispersal and gene flow (see [1]). Between-clade distances for 16S and COI range from 2.2 to 4.2%, with a proportion of interclade differentiation of 81.3%.

Our results are in agreement with previous estimates based on allozymes reporting considerable differentiation (mean genetic distance of 12.6%, [37]) and substructuring (mean *F*_ST_ = 0.24, [38]) between geographic populations of the *A. franciscana* group. For South American *A. franciscana* populations, mean *F*_ST_ values range from 0.38 (allozymes, [39]) to 0.91 (16S RFLPs, [40]). Marked genetic distances have also been reported between Mexican and North American *A. franciscana* [41]. In a study of the intraspecific phylogeography of the Mediterranean *A. salina* [42], COI distances between higher-level clades (as determined by nested clade phylogeographic analysis) ranged from 1.1 to 2.7%. The authors found a pattern of extensive regional endemism, suggestive of an early Pleistocene split of Mediterranean lineages and subsequent population isolation in a number of glacial refugia. Analogous interpretations have also been advanced to explain the presence of localized distinct phylogroups in the rotifer *Brachionus plicatilis* (mean COI divergence 2.8%) from the Iberian Peninsula [43] and the freshwater cladoceran *Daphnia magna* (COI divergences 0.73–1.74%) from Europe [13].

Our mitochondrial DNA data point to a similar pattern of regional endemism and phylogeographic structure at the microscale, typically seen in continental zooplankters [43]. The substantial amount of genetic differentiation between phylogroups, the support of monophyly of clades, and the absence of co-distributed lineages reflect accumulation of mutational differences following population separation. In other words, the lineage sorting process has been completed and the mitochondrial genealogy conveys a strong signal of historical fragmentation. Alternatively, gene flow between proximate populations may have been severely restricted in contemporary time, a possibility which can be effectively evaluated with additional and more extensive sampling.

Informative insights to the observed regional patterns of lineage endemism may be provided by consideration of biogeographic data pertinent to South America and, in particular, to the recent geological history of the Andes mountain range. Recent data indicate that the final phase of the central Andean uplift took place between 6.0 and 2.7 mya [44] affecting the diversification of taxa on either side of the mountain range [45]. Divergence rates for snapping shrimp COI sequences have been calculated at 1.4% per million years [46] and useful approximations are allowed when sequence divergences are in the order of <8%. Implementation of the above COI rate yields an estimated time of divergence between the most distantly related mitochondrial clades (clades 2 and 3, 4.2%) at 3.0 mya which is just before the last uplift of the Andes, in the late Pliocene. This is a strong indication that the Andean orogeny had a major effect on lineage splitting between clades 2 and 3 and extant populations have probably descended from gene pools which persisted through the recent periods of repeated glaciations. Thus, our results imply a longer demographic history than that expected by populations evolving during postglacial times. This is a signature of potential isolated refugial areas on both slopes of the Andes [45]. A rather similar pattern of spatial structuring of distinct genetic lineages has also been observed for *A. persimilis* populations on either side of the Andes [11]. Although such interpretations may involve a large margin of error, they provide an evolutionary timescale of lineage separations on which, given additional data, a more statistical phylogeographic framework can be devised (see [47,48]).

### 3.2. Nuclear Genealogy

The most salient feature of the inferred phylogeny is the incomplete lineage sorting of nuclear genes. Under neutrality, the expected time for lineage sorting to reciprocal monophyly is fourfold longer for nuclear lineages than for mitochondrial lineages [2]. Thus, due to a fourfold larger effective population size, the coalescent process proceeds more slowly for alleles at nuclear genes than for mitochondrial haplotypes. The nuclear tree quite clearly captures a snapshot of the above process for the ITS1 and p26 allelic lineages. In addition, due to the stochastic nature of genetic drift, its effects, simply by chance, may have not been registered strongly in the investigated nuclear loci. The dynamics of the specific sequences may also be involved by virtue of their being influenced by different forces, as ITS1 is a spacer region and p26 an intron region.

The incomplete sorting of ancestral polymorphisms is most apparent for the 1500 bp group of p26 alleles, and particularly for 1500-B (see Figure 2), which shows higher sequence diversity (0.4–14.8%) compared with the other groups of allelic lineages (1400, 2000) and contains representatives of all the respective mitochondrial clades. This paraphyletic pattern, as well as the subtopologies within the other groups (1400, 1500-A), may have likely arisen as a result of male-mediated dispersal. This mechanism erodes geographic structure in nuclear genes, yet leaves the mitochondrial DNA phylogeny unaffected (see [49]). It is also worth noting that the 1500-B allelic lineage is represented by populations with the highest proximity, a fact relating to the previous dispersal scenario. Moreover, allozyme analyses of South American *A. franciscana* have identified the populations of LVI and IQU as the most divergent ones, bearing a mixture of both private and shared polymorphisms [39]. An obvious critical requirement for the dispersal mechanism is that transported cysts developing into females do not reproduce with resident males. Although there is currently no evidence for intraspecific directionality in reproduction, clues may be obtained from the interspecific level where such a mechanism seems to work [12]. Together, these findings support the view that gene flow has been operating to such a degree as to prevent genetic differentiation by genetic drift or, alternatively, selection has favoured the maintenance of certain alleles in South American sites. It is interesting to see that populations CON and CEJ (together with GSL from North America) display a pattern of polymorphism for the p26 allelic lineages. In particular, all three p26 alleles are found in the CON population (1400, 1500, 2000) whereas for CEJ and GSL two p26 alleles were detected (1500, 2000) with the 1500 alleles grouped in different lineages for each population (see Figure 2). Although additional data are needed to explain the distribution of this polymorphism, it could be tentatively ascribed to the fact that the three populations (members of distinct mitochondrial clades, see Figure 1) represent the most ancestral stocks (possibly refugial sites) from which lineage colonization and subsequent random loss occurred.

Irrespective of the specific etiology for the observed nuclear genealogy, the overall phylogeographic evidence in the investigated *A. franciscana* populations supports a genealogical phase at the interval between gene splitting and population fragmentation. Put differently, this interval is where speciation occurs. The mitochondrial DNA evidence upholds a specific demographic independence between sets of *A. franciscana* populations. This is registered in the presence of reciprocally monophyletic clades, being, by definition, genetically distinct and free of exchange. Depending on the evolutionary timescale, these population sets may qualify as evolutionary significant units [50], thus justifying to a large extent arguments for incipient speciation in *A. franciscana*. Our data come to reinforce previous evidence on significant population differentiation in *A. franciscana*. A distinct correlation between chromocentre frequency and latitude, certain types of aneuploidy as registered in fluctuating chromosome numbers, extreme examples of ecological isolation, and an association between habitat type and heterozygosity in *A. franciscana* populations [23] are all conducive to lineage diversification and suggest the presence of both geographical and ecological forms. However, before solid statements on the possible superspecies status of *A. franciscana* are made, more thorough phylogeographic nuclear sequence assays, accounting for the temporal and spatial aspects of population genealogical structure, are needed in order to unite the observed genetic attributes of the species with its postulated past biogeography.

## 4. Experimental Section

### 4.1. Populations Analyzed and Data Assembly

**Figure 3.**
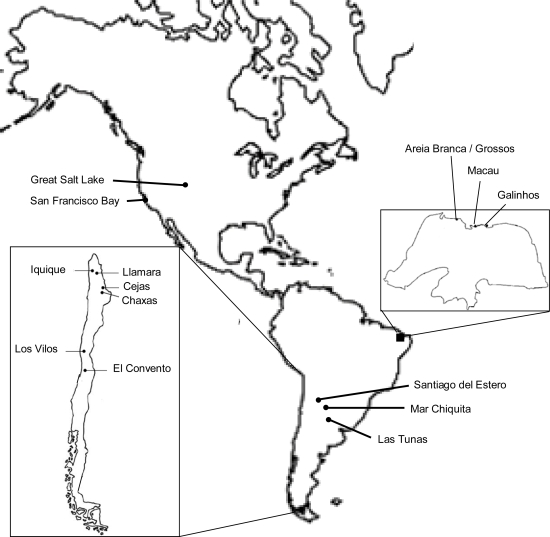
Map showing the locations of the *Artemia franciscana* populations analyzed.

We analyzed 14 *A. franciscana* populations from North (USA) and South America (Brazil, Chile, Argentina) (see Figure 3, Table 2). The three Brazilian feral populations originate from material inoculated more than 30 years ago in the respective sites [28]. We used published ITS1 and 16S rRNA sequences from Baxevanis *et al*. [11] and Kappas *et al*. [12] and also obtained novel sequences of these markers following the protocols by Kappas *et al*. [12] (for GenBank accession numbers see Table 1). Additionally, we amplified a 601 bp fragment of the mitochondrial cytochrome oxidase subunit I (COI) gene region and the entire second intron of the nuclear p26 gene. Whenever possible, we used the same individuals to those registered in GenBank for ITS1 and 16S rRNA.

For COI, a set of newly designed primers were employed (available upon request) based on deposited COI sequences of Anostraca. PCR conditions were: 4 min at 94 °C, 40 cycles of 50 sec at 94 °C, 50 sec at 50 °C, 50 sec at 72 °C and a final extension of 5 min at 72 °C. Total reaction volumes of 25 μL consisted of 2.5 μL template DNA, 5 μL 5 × PCR buffer, 2.5 mM MgCl_2_, 1 mM dNTPs, 0.001 mM of each primer and 1.25 U of *Taq* DNA polymerase (Expand High Fidelity^PLUS^ PCR System, Roche).

For p26, PCR conditions are detailed in Maniatsi *et al*. [29]. In all cases, bands were purified prior to direct sequencing (complementary strands). All amplifications were performed on a PTC-100^®^ Peltier thermal cycler (MJ Research). Sequencing reactions were electrophoresed on a PRISM 3730xl DNA analyzer (Applied Biosystems). The newly derived COI and p26 gene sequences were deposited in GenBank (see Table 1). Sequences were aligned using ClustalX 1.8 [30]. In all cases (16S, COI, ITS1, p26), genealogies were rooted using *A. salina* as outgroup (accession numbers: 16S: FJ007839, COI: GU248381, ITS1: DQ201303, p26: GU248407).

### 4.2. Sequence Analysis and Phylogenetic Reconstruction

Newly derived sequences were subjected to BLAST searches for confirmation of homology. For all data sets we conducted tests for homogeneity of base frequencies, substitution saturation, and presence of phylogenetic signal following the methods in Kappas *et al*. [12]. Values of genetic diversity within and distance between populations were computed in MEGA version 4 [31]. Corrected distances were used based on the best-fit substitution model as determined by Modeltest 3.7 [32].

Phylogenetic reconstruction was implemented in PAUP* 4.0b10 [33] under maximum parsimony. Phylogenetic inference was based on concatenated sequence data (mitochondrial: 16S + COI; nuclear: ITS1 + p26). Prior to data combination, conflicting phylogenetic signal was evaluated through Mantel tests on the corresponding genetic distance matrices. For each joint data set, trees were generated using heuristic searches with TBR (tree-bisection-reconnection) branch swapping and 500 random taxon additions. Nodal support was assessed by 1000 bootstrap replicates. Gaps were treated as missing data. Phylogenetic analysis was also conducted considering third codon positions only for COI.

For mitochondrial DNA, we evaluated isolation-by-distance (IBD) patterns by correlating geographic and genetic distances (corrected) between *A. franciscana* populations. We used the IBDWS version 3.14 (Isolation by Distance Web Service, [34]) to perform Mantel tests (both raw and log-transformed matrices, 30,000 randomizations) and Reduced Major Axis (RMA) regression analysis. We also tested for the presence of phylogeographic structure using DnaSP version 5 [35]. We used the nucleotide sequence-based *K*_ST_ statistic as given in Hudson *et al*. [36] in order to control for the presence of unique haplotypes in different groupings. Group assignment was based on the inferred topology during phylogenetic reconstruction. Statistical significance was assessed using a permutation test (10,000 randomizations).

## Figures and Tables

**Table 1.** List of *Artemia franciscana* individuals analyzed and GenBank accession numbers of sequences.

**Individuals**	**16S**	**COI**	**ITS1**
SFB8	FJ007826^a^	X69067	DQ201292^b^
GSL1	FJ007825^a^	GU248372	DQ201300^b^
LVI6, LVI7	FJ007833^a^, FJ007834^a^	GU248379, GU248380	-, FJ004929^a^
CEJ6, CEJ7	FJ007831^a^, FJ007832^a^	GU248375, GU248376	FJ004925^a^, FJ004935^a^
CHA6, CHA8	FJ007829^a^, FJ007830^a^	GU248377, GU248378	FJ004928^a^, FJ004926^a^
LLA6, LLA7	FJ007820^a^, FJ007821^a^	GU248365, GU248366	-, FJ004939^a^
CON6, CON9	FJ007823^a^, FJ007822^a^	GU248363, GU248364	FJ004941^a^, FJ004942^a^
IQU1, IQU8	FJ007827^a^, FJ007828^a^	GU248373, GU248374	DQ201296^a^, FJ004927^a^
MAC13	GU248382	GU248371	GU252102
ABG20	GU248383	GU248369	GU252103
GAL22	GU248384	GU248370	GU252104
CHI21	GU248385	GU248362	GU252105
LTU19	GU248386	GU248368	GU252106
SET3	GU248387	GU248367	GU252107
**p26**			
2000 bp	1500 bp		1400 bp

GSL_A^c^	SFB^c^	CON13B (GU248398)	LLA7 (GU248402)
CEJ5 (GU248389)	SFB10 (GU248391)	CON7A (GU248397)	CON7B (GU248401)
CON13A (GU248388)	GSL_B^c^	IQU3 (GU248396)	CON10 (GU248405)
	LVI8 (GU248393)	MAC13 (GU248406)	CHI21 (GU248404)
	CEJ9 (GU248399)	ABG20 (GU248392)	LTU13 (GU248400)
	CEJ1 (GU248395)	GAL22 (GU248390)	SET15 (GU248403)
	CHA9 (GU248394)		

a: [12];

b: [11];

c: [29]; Underlined sequences were obtained in this study.

**Table 2.** List of *Artemia franciscana* populations analyzed.

**Population** **(abbreviation)**	**Country**	**Geographic coordinates**
San Francisco Bay (SFB)	USA	37°28′N 122°30′W
Great Salt Lake (GSL)	USA	40°59′N 112°24′W
Macau (MAC)	Brazil	5°06′S 36°38′W
Areia Branca/Grossos (ABG)	Brazil	4°57′S 37°08′W
Galinhos (GAL)	Brazil	5°07′S 36°23′W
Los Vilos (LVI)	Chile	31°58′S 71°25′W
Cejas (CEJ)	Chile	23°02′S 68°13′W
Chaxas (CHA)	Chile	22°47′S 67°58′W
Llamara (LLA)	Chile	21°18′S 69°37′W
El Convento (CON)	Chile	33°52′S 71°44′W
Iquique (IQU)	Chile	20°40′S 70°15′W
Mar Chiquita (CHI)	Argentina	30°39′S 62°36′W
Las Tunas (LTU)	Argentina	33°45′S 62°19′W
Santiago del Estero (SET)	Argentina	27°21′S 64°13′W

## References

[1] Avise JC Phylogeography: The History and Formation of Species2nd Ed Harvard University Press Cambridge, MA, USA 2000

[2] Avise JC Molecular Markers, Natural History, and Evolution2nd Ed Sinauer Associates, Inc Sunderland, MA, USA 2004

[3] Bernardi G Sordino P Powers DA Concordant mitochondrial and nuclear DNA phylogenies for populations of the teleost fish *Fundulus heteroclitus* Proc. Natl. Acad. Sci. USA 1993 90 9271 9274 8105474 10.1073/pnas.90.20.9271 PMC47549

[4] Burton RS Lee B-N Nuclear and mitochondrial gene genealogies and allozyme polymorphism across a major phylogeographic break in the copepod *Tigriopus californicus* Proc. Natl. Acad. Sci. USA 1994 91 5197 5201 7910968 10.1073/pnas.91.11.5197 PMC43959

[5] Willett CS Ladner JT Investigations of fine-scale phylogeography in *Tigriopus californicus* reveal historical patterns of population divergence BMC Evol. Biol 2009 9 139 19549324 10.1186/1471-2148-9-139 PMC2708153

[6] Mills S Lunt DH Gómez A Global isolation by distance despite strong regional phylogeography in a small metazoan BMC Evol. Biol 2007 7 225 17999774 10.1186/1471-2148-7-225 PMC2254418

[7] Adamowicz SJ Petrusek A Colbourne JK Hebert PDN Witt JDS The scale of divergence: A phylogenetic appraisal of intercontinental allopatric speciation in a passively dispersed freshwater zooplankton genus Mol. Phylogenet. Evol 2009 50 423 436 19124080 10.1016/j.ympev.2008.11.026

[8] Gajardo GM Sorgeloos P Beardmore JA Inland hypersaline lakes and the brine shrimp *Artemia* as simple models for biodiversity analysis at the population level Saline Syst 2006 2 14 17132175 10.1186/1746-1448-2-14 PMC1684253

[9] Green AJ Sánchez MI Amat F Figuerola J Hontoria F Ruiz O Hortas F Dispersal of invasive and native brine shrimps *Artemia* (Anostraca) *via* waterbirds Limnol. Oceanogr 2005 50 737 742

[10] Mayr E Animal Species and Evolution Belknap Press Cambridge, MA, USA 1963

[11] Baxevanis AD Kappas I Abatzopoulos TJ Molecular phylogenetics and asexuality in the brine shrimp *Artemia* Mol. Phylogenet. Evol 2006 40 724 738 16753307 10.1016/j.ympev.2006.04.010

[12] Kappas I Baxevanis AD Maniatsi S Abatzopoulos TJ Porous genomes and species integrity in the branchiopod *Artemia* Mol. Phylogenet. Evol 2009 52 192 204 19306934 10.1016/j.ympev.2009.03.012

[13] de Gelas K de Meester L Phylogeography of *Daphnia magna* in Europe Mol. Ecol 2005 14 753 764 15723667 10.1111/j.1365-294X.2004.02434.x

[14] King JL Hanner R Cryptic species in a “living fossil” lineage: Taxonomic and phylogenetic relationships within the genus*Lepidurus* (Crustacea: Notostraca) in North America Mol. Phylogenet. Evol 1998 10 23 36 9751915 10.1006/mpev.1997.0470

[15] Lee CE Global phylogeography of a cryptic copepod species complex and reproductive isolation between genetically proximate “populations” Evolution 2000 54 2014 2027 11209778 10.1111/j.0014-3820.2000.tb01245.x

[16] Witt JDS Hebert PDN Cryptic species diversity and evolution in the amphipod genus *Hyalella* within central glaciated North America: A molecular phylogenetic approach Can. J. Fish. Aquat. Sci 2000 57 687 698

[17] Gómez A Serra M Carvalho GR Lunt DH Speciation in ancient cryptic complexes: Evidence from the molecular phylogeny of *Brachionus plicatilis* (Rotifera) Evolution 2002 56 1431 1444 12206243 10.1111/j.0014-3820.2002.tb01455.x

[18] Papakostas S Triantafyllidis A Kappas I Abatzopoulos TJ The utility of the 16S gene in investigating cryptic speciation within the *Brachionus plicatilis* species complex Mar. Biol 2005 147 1129 1139

[19] De Meester L Gómez A Okamura B Schwenk K The Monopolization Hypothesis and the dispersal–gene flow paradox in aquatic organisms Acta Oecol 2002 23 121 135

[20] Triantaphyllidis GV Abatzopoulos TJ Sorgeloos P Review of the biogeography of the genus *Artemia* (Crustacea, Anostraca) J. Biogeogr 1998 25 213 226

[21] van Stappen G Zoogeography Artemia: Basic and Applied Biology1st Ed Abatzopoulos TJ Beardmore JA Clegg JS Sorgeloos P Kluwer Academic Publishers Dordrecht, The Netherlands 2002 171 224

[22] Lenz PH Ecological studies on *Artemia*: A review Artemia Research and Its Applications. Ecology, Culturing, Use in Aquaculture Sorgeloos P Bengtson DA Decleir W Jaspers E Universa Press Wetteren, Belgium 1987 3 5 18

[23] Abatzopoulos TJ Beardmore JA Clegg JS Sorgeloos P Artemia: Basic and Applied Biology1st Ed Kluwer Academic Publishers Dordrecht, The Netherlands 2002

[24] Abatzopoulos TJ Kappas I Bossier P Sorgeloos P Beardmore JA Genetic characterization of *Artemia tibetiana* (Crustacea: Anostraca) Biol. J. Linn. Soc 2002 75 333 344

[25] Lenz PH Browne RA Ecology of *Artemia* Artemia Biology1st Ed Browne RA Sorgeloos P Trotman CAN CRC Press Boca Raton, FL, USA 1991 237 253

[26] Kappas I Abatzopoulos TJ Van Hoa N Sorgeloos P Beardmore JA Genetic and reproductive differentiation of *Artemia franciscana* in a new environment Mar. Biol 2004 146 103 117

[27] Bowen ST Fogarino EA Hitchner KN Dana GL Chow VHS Buoncristiani MR Carl JR Ecological isolation in *Artemia*: Population differences in tolerance of anion concentrations J. Crust. Biol 1985 5 106 129

[28] Camara MR Dispersal of *Artemia franciscana* Kellogg (Crustacea; Anostraca) populations in the coastal saltworks of Rio Grande do Norte, northeastern Brazil Hydrobiologia 2001 466 145 148

[29] Maniatsi S Baxevanis AD Abatzopoulos TJ The intron 2 of p26 gene: A novel genetic marker for discriminating the two most commercially important *Artemia franciscana* subspecies J. Biol. Res. (Thessalon.) 2009 11 73 82

[30] Thompson JD Gibson TJ Plewniak F Jeanmougin F Higgins DG The ClustalX windows interface: Flexible strategies for multiple sequence alignment aided by quality analysis tools Nucleic Acids Res 1997 25 4876 4882 9396791 10.1093/nar/25.24.4876 PMC147148

[31] Tamura K Dudley J Nei M Kumar S MEGA4: Molecular Evolutionary Genetics Analysis (MEGA) software version 4.0 Mol. Biol. Evol 2007 24 1596 1599 17488738 10.1093/molbev/msm092

[32] Posada D Crandall KA Modeltest: Testing the model of DNA substitution Bioinformatics 1998 14 817 818 9918953 10.1093/bioinformatics/14.9.817

[33] Swofford DL PAUP* Phylogenetic Analysis Using Parsimony (* and Other Methods) Version 4 Sinauer Associates Sunderland, MA, USA 1998

[34] Jensen JL Bohonak AJ Kelley ST Isolation by distance, web service BMC Genet 2005 6 13 15760479 10.1186/1471-2156-6-13 PMC1079815

[35] Librado P Rozas J DnaSP v5: A software for comprehensive analysis of DNA polymorphism data Bioinformatics 2009 25 1451 1452 19346325 10.1093/bioinformatics/btp187

[36] Hundson RR Boos DD Kaplan NL A statistical test for detecting geographic subdivision Mol. Biol. Evol 1992 9 138 151 1552836 10.1093/oxfordjournals.molbev.a040703

[37] Abreu-Grobois FA Population Genetics of ArtemiaPh.D. Thesis; University of Wales: Swansea, UK, 1983

[38] Beardmore JA Abreu-Grobois FA Taxonomy and evolution in the brine shrimp *Artemia* Protein Polymorphism: Adaptive and Taxonomic Significance Oxford GS Rollinson D Academic Press London, UK 1983 153 164

[39] Gajardo G Da Conceicao M Weber L Beardmore JA Genetic variability and interpopulational differentiation of *Artemia* strains from South America Hydrobiologia 1995 302 21 29

[40] Gajardo G Crespo J Triantafyllidis A Tzika A Baxevanis AD Kappas I Abatzopoulos TJ Species identification of Chilean *Artemia* populations based on mitochondrial DNA RFLP analysis J. Biogeogr 2004 31 547 555

[41] Tizol-Correa R Maeda-Martínez AM Weekers PHH Torrentera L Murugan G Biodiversity of the brine shrimp *Artemia* from tropical salterns in southern México and Cuba Current Sci 2009 96 81 87

[42] Muñoz J Gómez A Green AJ Figuerola J Amat F Rico C Phylogeography and local endemism of the native Mediterranean brine shrimp *Artemia salina* (Branchiopoda: Anostraca) Mol. Ecol 2008 17 3160 3177 18510585 10.1111/j.1365-294X.2008.03818.x

[43] Gómez A Carvalho GR Lunt DH Phylogeography and regional endemism of a passively dispersing zooplankter: mtDNA variation of rotifer resting egg banks Proc. R. Soc. Lond. B 2000 267 2189 2197 10.1098/rspb.2000.1268 PMC1690794 11413632

[44] Gregory-Wodzicki KM Uplift history of the Central and Northern Andes: A review Geol. Soc. Am. Bull 2000 112 1091 1105

[45] Ramírez CC Salazar M Palma RE Cordero C Mezabasso L Phylogeographical analysis of neotropical *Rhagoletis* (Diptera: Tephritidae): Did the Andes uplift contribute to current morphological differences? Neotrop. Entomol 2008 37 651 661 19169552 10.1590/s1519-566x2008000600005

[46] Knowlton N Weigt LA New dates and new rates for divergence across the Isthmus of Panama Proc. R. Soc. Lond. B 1998 265 2257 2263

[47] Templeton AR Nested clade analyses of phylogeographic data: Testing hypotheses about gene flow and population history Mol. Ecol 1998 7 381 397 9627999 10.1046/j.1365-294x.1998.00308.x

[48] Templeton AR Using phylogeographic analyses of gene trees to test species status and processes Mol. Ecol 2001 10 779 791 11298987 10.1046/j.1365-294x.2001.01199.x

[49] Palumbi SR Baker SC Contrasting population structure from nuclear intron sequences and mtDNA of humpback whales Mol. Biol. Evol 1994 11 426 435 7912407 10.1093/oxfordjournals.molbev.a040115

[50] Ryder OA Species conservation and the dilemma of subspecies Trends Ecol. Evol 1986 1 9 10 10.1016/0169-5347(86)90035-2 21227791

